# Soft Tissue Pseudomyogenic Hemangioendothelioma in the Buttock: A Case Report

**DOI:** 10.2174/0115734056433778251217073245

**Published:** 2026-01-30

**Authors:** Bokdong Yeo, Yu Sung Yoon, Mee-Seon Kim

**Affiliations:** 1 Department of Radiology, Kyungpook National University Hospital, Daegu, South Korea; 2 Department of Radiology, School of Medicine, Kyungpook National University, Kyungpook National University Hospital, Daegu, South Korea; 3 Department of Pathology, School of Dentistry, Kyungpook National University, Kyungpook National University Hospital, Daegu, South Korea

**Keywords:** Pseudomyogenic hemangioendothelioma, Epithelioid sarcoma-like hemangioendothelioma, Soft tissue neoplasm, Magnetic resonance imaging

## Abstract

**Introduction:**

Pseudomyogenic Hemangioendothelioma (PMHE), also known as epithelioid sarcoma-like hemangioendothelioma, is a rare, indolent, low-grade vascular tumor. It typically presents as firm cutaneous nodules, with a predilection for the lower extremities and a male predominance. While numerous cases have been reported in pathology literature, detailed radiologic descriptions, particularly of soft tissue origins, are scarce. This report aims to bridge this gap by presenting a rare case of PMHE with comprehensive imaging findings.

**Case Presentation:**

We report on a 67-year-old male who presented with painful, palpable papules on his right buttock. MRI revealed multifocal dermal nodules demonstrating low signal intensity on T1-weighted images and high signal intensity with a distinctive peripheral high-signal halo on T2-weighted images. Notably, T1 gadolinium fat-saturated sequences exhibited marked enhancement with a characteristic peripheral rim enhancement pattern. The lesions were confined to the cutaneous layer. Initial radiological differentials included post-inflammatory granuloma and sarcoma. Histopathological examination confirmed PMHE. PET/CT demonstrated no evidence of systemic metastasis, and the patient has remained recurrence-free for two years following surgery.

**Conclusion:**

This report highlights a rare case of cutaneous PMHE and details its distinctive MRI features, particularly the peripheral rim enhancement. Given its rarity and often non-specific clinical and imaging presentations, there is a significant potential for misdiagnosis. Therefore, it is crucial for radiologists to be aware of PMHE and familiarize themselves with its characteristic radiological patterns to facilitate accurate, timely diagnosis and ensure appropriate patient management.

## 1 INTRODUCTION

Pseudomyogenic hemangioendothelioma (PMHE), also known as epithelioid sarcoma-like hemangioendothelioma, is a rare, indolent, low-grade vascular tumor. Originally described by Mirra *et al.* in 1992 as a fibroma-like variant of epithelioid sarcoma, its clinicopathological features were later clarified by Hornick and Fletcher in 2011, leading to its current designation as PMHE [1, 2]. The term “pseudomyogenic” refers to its histopathologic resemblance to myoid and epithelioid tumors.

PMHE typically presents as one or multiple ill-defined, firm cutaneous nodules, with a predilection for the lower extremities [3]. Lesions may be asymptomatic or painful and may involve various anatomical sites, including the dermis, subcutis, bone, and muscle. It has a marked male predilection (M: F ≈ 5:1), with most patients between the second and fifth decades of life [2].

Although PMHE has been characterized in the surgical and pathology literature, radiologic descriptions-especially those involving soft-tissue lesions-remain scarce, with only small case series and isolated case reports available to date [4-7]. This study aims to address this gap by presenting a rare case of PMHE arising in the buttocks of a 67-year-old male who presented with painful papules that had persisted for six months. The diagnosis of pseudomyogenic hemangioendo-thelioma was confirmed pathologically, and characteristic rim enhancement was observed on MRI.

## 2 CASE PRESENTATION

**Fig. (1):**
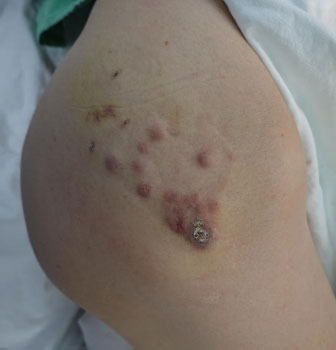
Multiple bean-sized erythematous, firm papules on the right buttock of a 67-year-old man.

**Fig. (2):**
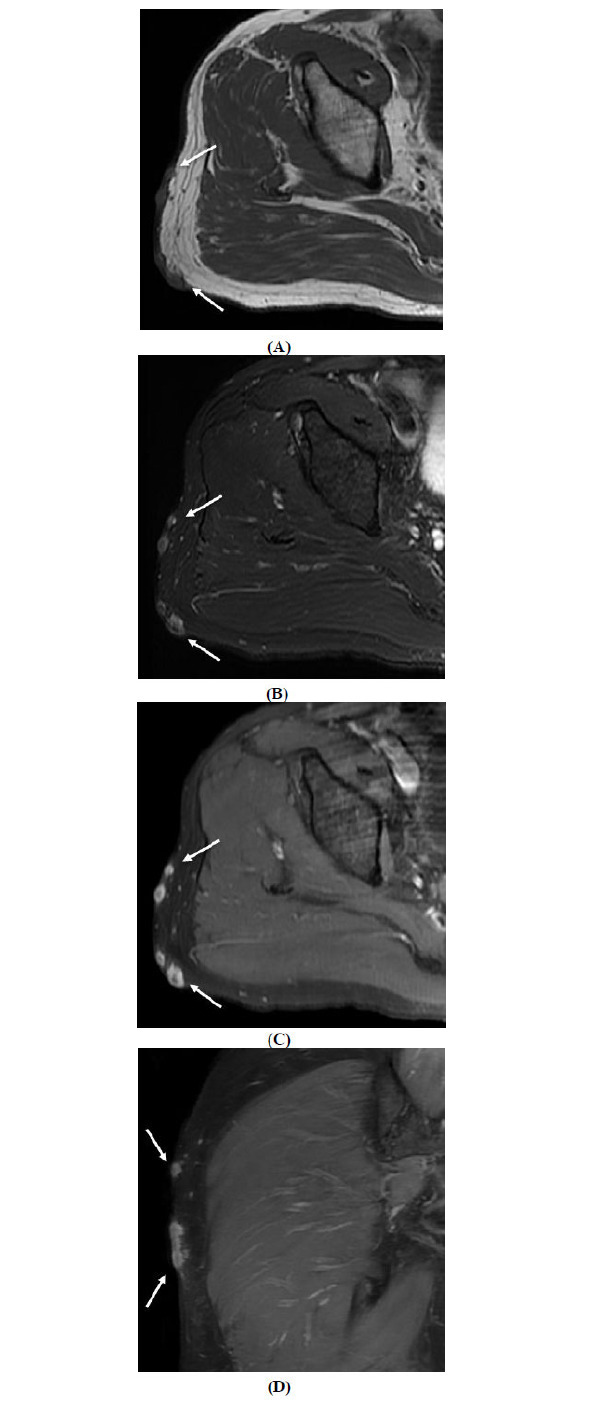
(**A**) Axial T1-weighted, (**B**) axial T2 fat-saturated, (**C**) axial gadolinium-enhanced T1 fat-saturated, and (**D**) coronal gadolinium-enhanced T1 fat-saturated MRI images show multiple cutaneous nodules (white arrows) on the right buttock. The nodules demonstrate low signal intensity on T1-weighted images and high signal intensity with a distinctive peripheral halo on T2-weighted images, with a characteristic peripheral rim enhancement pattern on contrast-enhanced sequences.

**Fig. (3):**
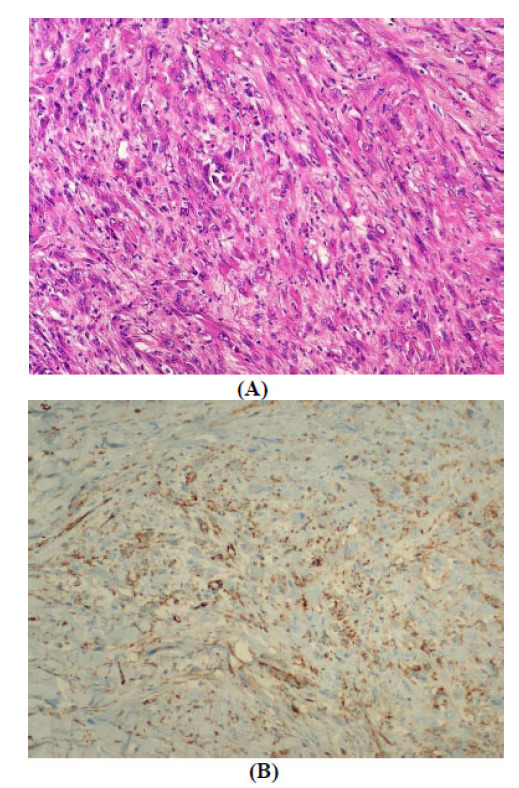
Histopathologic findings of pseudomyogenic hemangioendothelioma. (**A**) Atypical epithelioid-to-spindle cell proliferation, characterized by prominent nucleoli and eosinophilic plump cytoplasm, bears resemblance to rhabdomyoblasts, along with stromal neutrophils.(H&E, x200) (**B**) The tumor exhibits positivity for the CD31 protein.(CD31, x200).

**Fig. (4):**
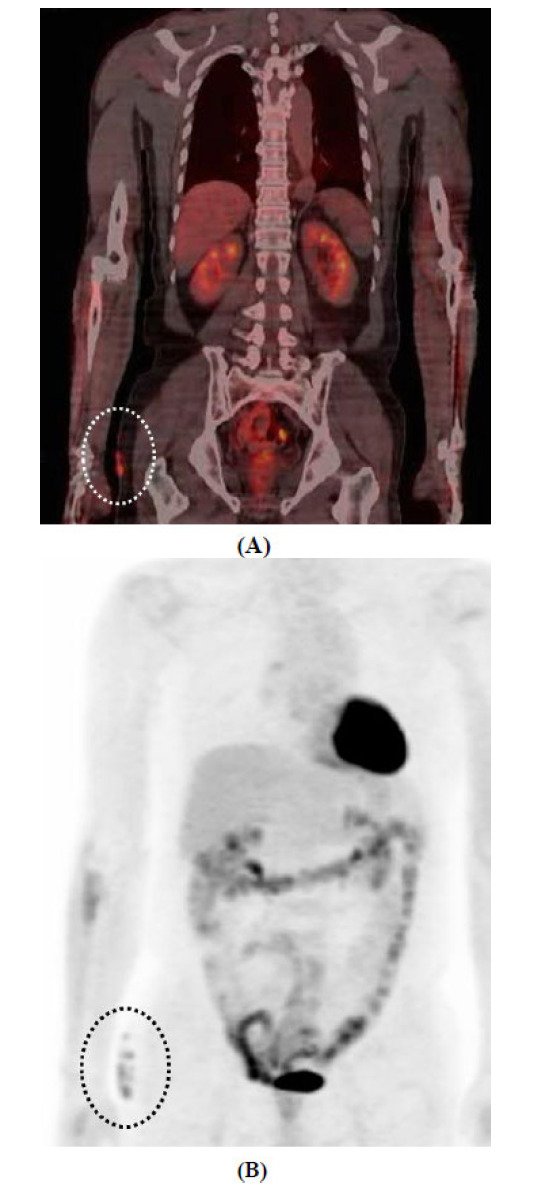
(**A**) Fused PET/CT image with a white dotted circle highlights the clustered hypermetabolic nodules in the dermal layer of the right pelvis. (**B**) Maximum intensity projection (MIP) PET/CT image with a black dotted circle demonstrates the corresponding hypermetabolic lesions.

A 67-year-old male presented to our dermatology outpatient clinic with palpable, bean-sized, erythematous, and painful papules on his right buttock, extending approximately 8 cm in total (Fig. **1**). He had no significant medical history other than hypertension and diabetes mellitus. The symptoms and skin lesions had appeared approximately six months prior to his clinic visit. To assess the characteristics and extent of the lesions, pelvic MRI was performed and revealed multiple subcentimeter dermal nodular lesions at the lateral aspect of the right buttock, accompanied by perilesional dermal thickening. The nodules showed low signal intensity on T1-weighted images and high signal intensity with a distinctive peripheral high-signal halo on T2-weighted images (Fig. **2A** and **B**). The lesions were confined to the cutaneous layer, with no evidence of bone involvement. On T1-weighted gadolinium-enhanced fat-saturated images, the lesions demonstrated a marked peripheral rim enhancement pattern (Fig. **2C** and **D**). Based on these findings, a post-inflammatory soft-tissue granuloma was suspected, with sarcoma considered a less likely differential diagnosis. Following radiological evaluation, a complete surgical excision biopsy of all skin lesions, including the surrounding dermal tissue, was performed with a lateral safety margin of 4 mm and a basal safety margin of 1 mm. The histopathologic examination confirmed the diagnosis of PMHE (Fig. **3A** and **B**). To evaluate potential systemic involvement, PET/CT was performed and revealed no evidence of metastasis (Fig. **4A** and **B**). Following surgical excision, the patient was placed under clinical surveillance for two years, during which no recurrence was observed at the last follow-up.

## 3 DISCUSSION

Herein, we present a rare case of pseudomyogenic hemangioendothelioma (PMHE) of the lower extremity, highlighting its characteristic imaging findings and emphasizing the importance of multidisciplinary correlation for accurate diagnosis. This case adds to the limited body of literature on PMHE, particularly regarding its radiologic features, which can often be non-specific and mimic more common soft-tissue tumors. Understanding the key clinical, pathological, and radiologic aspects of PMHE is crucial for clinicians, given its distinct genetic signature and propensity for local recurrence.

Hornick and Fletcher [2] examined 50 cases of the distinctive tumor type. Their findings indicated a strong male predominance (almost 5:1), with nearly all patients between the second and fifth decades of life. The majority of tumors (78%) originated in the extremities, frequently presenting as multifocal disease involving several tissue planes. The dermis and subcutis were the most commonly affected sites, with nearly half of the patients exhibiting intramuscular nodules and 20% demonstrating intraosseous lesions.

The precise pathogenesis of PMHE remains unclear. Recently, a balanced translocation t(7;19)(q22;q13) was identified in more than 96% of PMHE cases [4], resulting in a SERPINE1-FOSB fusion. This fusion may possess oncogenic potential by exploiting the SERPINE1 promoter, which is highly expressed in endothelial cells. Consequently, it leads to elevated FOSB expression, a component of the activator protein-1 complex implicated in cellular processes such as proliferation, differentiation, and apoptosis [8]. In immunohistochemistry, FOSB positivity is a valuable marker for diagnosing PMHE, given its association with the initial genetic alteration [9].

In the present case, the histologic features of the PMHE were consistent with previous reports, demonstrating atypical epithelioid-to-spindle cell proliferation with prominent nucleoli and eosinophilic cytoplasm, resembling rhabdomyoblasts. The tumor consisted of loose fascicles of spindle or oval cells with elongated nuclei and stromal neutrophilic infiltration. These findings align with recent clinicopathologic descriptions of PMHE [10]. The term “pseudomyogenic” reflects the histologic resemblance to both myoid and epithelioid tumors. Giant cells were also frequently observed. Rhabdomyoblast-like cells may help differentiate PMHE from osteoblastoma and giant cell tumors. Despite its vascular origin, PMHE lacks a local vasoformative architecture and usually resembles epithelioid angiosarcoma. Immunohistochemistry is essential for differentiating PMHE from other tumors, particularly epithelioid sarcoma [11].

In the present case, PMHE demonstrated low signal intensity on T1-weighted sequences and high signal intensity with a distinctive peripheral high-signal halo on fat-saturated T2-weighted images. On gadolinium-enhanced fat-saturated images, the lesions demonstrated marked enhancement with a characteristic peripheral rim pattern. Although it is challenging to distinguish PMHE solely on imaging, the radiologic differential diagnosis includes undifferentiated pleomorphic sarcoma (UPS), dermatofibrosarcoma protuberans (DFSP), and primary cutaneous lymphoma. A wide range of tumors affecting the cutaneous layer, presenting as discrete nodules, should be considered in the radiologic differential diagnosis.

First, undifferentiated pleomorphic sarcoma (UPS)-the most common type of soft tissue sarcoma-occurs in approximately half of cases in the lower extremities and typically demonstrates intermediate-to-low signal intensity similar to adjacent muscle on T1-weighted images, intermediate-to-high signal intensity on T2-weighted images, and prominent enhancement of solid components. However, the UPSs are typically large, well-circumscribed masses, usually exceeding 5 cm in diameter [12, 13]. They are also associated with significantly increased fluorodeoxyglucose (FDG) uptake on PET/CT compared with low-grade tumors such as PMHE. Indeed, retrospective PET/CT studies in soft tissue sarcomas have shown that high-grade sarcomas, including UPS, correlate with markedly higher maximum standardized uptake values (SUVmax) [14].

Dermatofibrosarcoma protuberans (DFSP) is a low-grade malignant tumor arising from dermal and subcutaneous tissues and represents the most common form of cutaneous sarcoma. The trunk and extremities are the most common sites of involvement. On MRI, DFSP appears as a well-defined, subcutaneous, multinodular mass with low signal intensity on T1-weighted images, high signal intensity on T2-weighted images, and enhancement [15].

Primary cutaneous lymphoma, a group of extranodal non-Hodgkin lymphomas, typically demonstrates nonspecific radiologic findings such as skin thickening with a nodular pattern or plaque-like configuration. FDG PET/CT almost invariably shows increased metabolic activity in these lesions [16].

In the present case, contrast-enhanced imaging demonstrated a peripheral rim enhancement pattern. Although no definite myxoid or fibromyxoid matrix was identified in our specimen, previous studies have reported that some PMHE cases contain areas of fibrous or myxoid stroma [17]. Based on these findings, we cautiously hypothesize that our lesion’s peripheral enhancement may reflect a matrix-associated mechanism, in which relatively hypercellular and vascular tumor peripheries enhance more strongly than central matrix-rich or inflamed zones. A similar stromal–vascular interplay has been described in epithelioid hemangioendothelioma, where enhancement varies according to the spatial distribution of tumor cells and fibromyxoid stroma [18]. By analogy, our findings may represent a comparable stromal-vascular process in PMHE. As the stromal composition could not be precisely correlated with imaging in this case, this interpretation is presented as a hypothesis-generating observation rather than a definitive imaging feature.

Differentiating this rare low-grade soft-tissue malignancy from other cutaneous neoplasms solely on clinical and radiological findings can be challenging, as highlighted by our initial differential diagnosis of post-inflammatory granuloma. The paramount risk of misdiagnosis lies in attributing the lesion to an inflammatory dermatosis or a benign condition, which delays pathological confirmation and definitive surgical intervention. This case involves a 67-year-old male, placing him in an age group (65 and older) that recent investigations have identified as high-risk for misdiagnosis, particularly in cases of skin malignancies [19]. The sources of misdiagnosis in such cases are often multifactorial: the non-specific clinical presentation (e.g., painful papules), the sheer rarity of PMHE leading to low clinical suspicion, and the radiological overlap with benign entities. Therefore, clinicians should maintain a high index of suspicion for palpable, painful masses refractory to conservative management, especially in elderly patients, and actively pursue timely histopathological diagnosis. It is crucial to avoid unplanned excision before histopathologic confirmation and accurate delineation of tumor boundaries through appropriate imaging studies.

Hornick and Fletcher [2] reported that approximately 60% of patients experienced local recurrence or development of additional nodules over time. Similarly, Yang *et al.* [10] reported a clinicopathologic cohort showing a comparable tendency toward recurrent or multifocal disease, emphasizing the importance of long-term surveillance in patients with PMHE. In the present case, the patient has been under clinical surveillance for two years following surgery, with no evidence of recurrence to date.

## CONCLUSION

This case demonstrates the characteristic peripheral rim enhancement of cutaneous PMHE on MRI and highlights the diagnostic challenges in elderly patients, who are at increased risk of misdiagnosis for skin malignancies. Radiologists should maintain heightened awareness of this rare entity to facilitate timely diagnosis and appropriate management.

## Data Availability

Not applicable.

## LIST OF ABBREVIATIONS

PMHE: Pseudomyogenic Hemangioendothelioma
FDG: Fluorodeoxyglucose
PET/CT: Positron Emission Tomography/Computed Tomography
MRI: Magnetic Resonance Imaging
CT: Computed Tomography
UPS: Undifferentiated Pleomorphic Sarcoma
DFSP: Dermatofibrosarcoma Protuberans
SUVmax: Maximum Standardized Uptake Value

## STANDARDS OF REPORTING

CARE guidelines were followed.
